# Reversible splenial lesion syndrome in neuroleptic malignant syndrome

**DOI:** 10.2349/biij.5.4.e24

**Published:** 2009-10-01

**Authors:** SA Al-Edrus, R Norzaini, R Chua, SD Puvanarajah, M Shuguna, S Muda

**Affiliations:** 1 Imaging Department, Universiti Putra Malaysia, Malaysia; 2 Radiology Department, Hospital Kuala Lumpur, Malaysia; 3 Neurology Department, Hospital Kuala Lumpur, Malaysia; 4 Radiology Department, Universiti Kebangsaan Malaysia, Malaysia

**Keywords:** corpus callosum, reversible splenial, neuroleptic malignant syndrome

## Abstract

**Background:**

Reversible focal lesions in the splenium of the corpus callosum (SCC) or reversible splenial lesion syndrome are rare and little is known about their pathophysiology.

**Case summary:**

The authors describe a case of a 65-year-old female who presented with fever, abnormal behaviour and mild hypernatremia. She was on neuropsychiatric treatment for bipolar disorder but denied any history of seizure. After an extensive workout to exclude infection, a clinical diagnosis of neuroleptic malignant syndrome (NMS) was made. Initial magnetic resonance imaging (MRI) of the brain showed a lesion in the SCC characterized by high-signal intensity on T2-weighted and FLAIR sequences with reduced signal intensity on T1-weighted sequence. Diffuse weighted imaging (DWI) showed restricted diffusion. There was no enhancement following Gadolinium administration. The follow-up MRI 8 weeks later showed complete resolution of the SCC lesion.

**Conclusion:**

While the pathophysiology of reversible SCC lesions is still unclear, this case highlights the need to consider NMS in the differential diagnosis of reversible splenial lesion of the corpus callosum.

## INTRODUCTION

Reversible focal lesions in the splenium of the corpus callosum (SCC) or reversible splenial lesion syndrome are rare and have only been described in recent years. It has been reported in a few cases of mild encephalitis and encephalopathy caused by various infective agents and has also been reported in less than 40 epilepsy patients on antiepileptic treatment. The underlying pathophysiology of these lesions still remains unclear and to this date, various postulations have been put forward as the probable cause. The authors present a case of this rare finding in a patient with NMS who was on neuropsychiatric treatment for bipolar disorder.

## CASE REPORT>

A 32-year-old woman presented with a 1-week history of fever, abnormal behaviour and refusal to eat and talk. She was a known case of bipolar disorder and was on multiple neuropsychiatric drugs: clomazapine 10 mg daily, lithium carbonate 300 mg (morning dose) and 600 mg (nocturnal dose), lorazepam 1 mg bd and 1.5 mg nocturnal dose prn and benzhexol 4 mg tds. On examination, she was conscious, flushed and able to open eyes spontaneously. Her pupils were equally reactive. There was no neck stiffness. Motor examination showed increased tone and brisk reflexes, more on the left side while the power was reduced to 3/5 in the upper limbs. She also had tremors on the right side. Plantar reflexes presented bilaterally.

Laboratory investigation showed leucocytosis with predominant neutrophilia and normal platelet and hemoglobin counts. Liver function test showed mild elevation of the alanine transaminase and alkaline phosphatase. Renal profile showed raised sodium level (161 mmol/l). Creatine phosphokinase (CPK) was markedly raised (537 U/l). Lumbar puncture was done and the opening pressure was raised (35 cm H_2_O). Cerebrospinal fluid examination did not show any presence of white cells. Ziehl-Nielsen stain, Gram stain and Indian ink stain were all negative. The levels of glucose and proteins were within normal limits. *Hemophilus influenza B*, *Streptococcus pneumoniae*, *Neisseria meningitides* or Streptococcus group B antigens were not detected in the cerebrospinal fluid.

**Figure 1:**
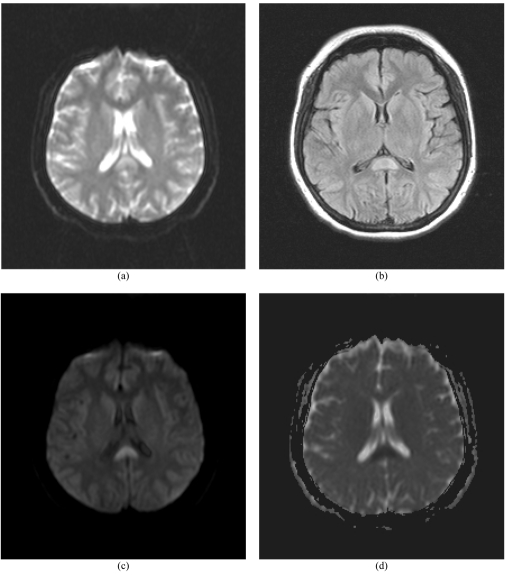
MRI imaging of the brain on admission with (a) axial T2-weighted, (b) axial FLAIR, (c) axial DWI and (d) corresponding ADC map. An ovoid lesion is seen at the centre of the splenium of the corpus callosum, hyperintense on T2 and FLAIR with markedly restricted diffusion and decreased ADC values.

CT scan of the brain showed a slightly swollen and hypodense splenium of the corpus callosum. Subsequent brain MRI showed swollen splenium of corpus callosum and appeared hypointense on T1-weighted image, hyperintense on T2-weighted image and FLAIR. Restriction in diffusion was observed in the DWI with decreased ADC values (Figure 1). No enhancement was noted on post-following gadolinium.

Her CPK level showed an up-going trend from the time of admission. It reached the maximum level of 1213 U/l on the 4^th^ day of admission and correspondingly, she was noted to be more aggressive verbally. She was empirically treated for possible CNS infection with intravenous ceftriaxone 1 g twice daily and C-Penicillin 2 megaunit 6-hourly. She was also put on a rehydration regime in view of the rising CPK levels and hypernatremia.

**Figure 2:**
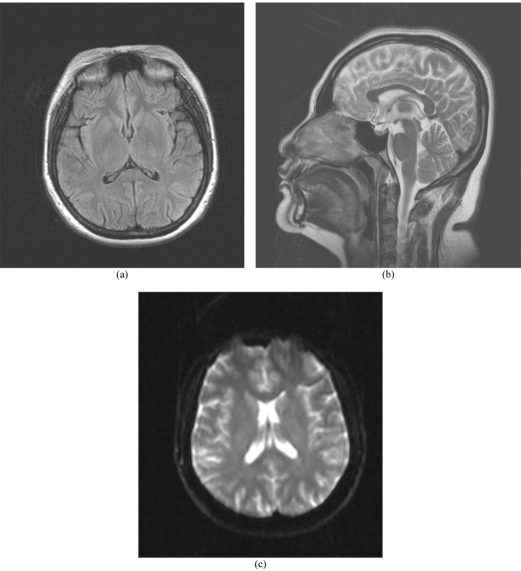
Follow up MR imaging performed 8 weeks after discharge with (a) axial FLAIR, (b) sagittal T2-weighted and (c) axial ADC map showing complete resolution of the SCC lesion and normalization of ADC values.

Her temperature returned to normal and both CPK and sodium levels came down to 417 U/l and 156 mmol/l, respectively. Upon discharge, there was only minimal cog-wheel rigidity. She was otherwise well and able to talk and eat as usual. A follow-up MRI done 8 weeks after discharge showed complete resolution of the splenial lesion (Figure 2). In view of the clinical history and presentation as well as the laboratory findings, a clinical diagnosis of neuroleptic malignant syndrome was made.

## DISCUSSION

Reversible splenial lesion of the corpus callosum has only been described in recent years and has been reported in only a few patients to date. The majority of reported cases have been found in patients with epilepsy undergoing antiepileptic treatment [1-4]. In these cases, different pathophysiologic hypotheses have been put forward as the possible etiology and among them are the transient effects of anti-epileptic drugs on arginine-vasopressin and its function in fluid balance systems in a condition of vitamin deficiency [1], reversible demyelination related to anti-epileptic drug toxicity [2] and transient focal oedema due to transcallosal seizure spread in secondary generalized seizures [3].

Another recently described clinicoradiological syndrome is the finding of a solitary reversible SCC lesion associated with mild encephalitis or encephalopathy [5-7]. Various causative agents have been attributed to this findings including influenza A, mumps virus, adenovirus, rotavirus, varicella-zoster virus, *Escherichia coli*, measles and *Salmonella enteritidis*.

In both these groups, the consistent finding is the similar signal characteristics of the SCC lesions on MRI. All lesions demonstrate hypointense signal on T1 and hyperintense signal on T2 and FLAIR sequences. Restricted diffusion was also seen in all cases that underwent DWI sequence. None of these lesions showed enhancement following gadolinium administration and complete resolution of the SCC lesion was seen in all patients on follow-up MRI.

In the group of patients with encephalitis/encephalopathy, complete resolution was seen on repeat imaging performed three days to two months following the abnormal study, regardless of the different causative agent. All reported cases had a mild clinical course with complete recovery seen within a month after onset of neurological symptoms. None of the patients developed permanent neurological sequelae.

In the patient described in this case report, both seizures and encephalitis were excluded. Thorough blood and cerebrospinal fluid examinations were also negative for encephalitis/encephalopathy. The diagnosis of NMS was made based on the positive history of neuropychiatric treatment as well as the clinical presentation and laboratory findings.

NMS is a relatively rare but potentially fatal side effect of antipsychotic medications, which was first described by Delay et al in 1960 during early trials of haloperidol. The incidence of NMS ranges between 0.02% to 3.23% of psychiatric patients receiving neuropsychiatric treatment. Clinically, it is characterized by an abnormal mental status, hyperthermia, ‘lead-pipe’ rigidity, akinesia or dystonia, autonomic instability, rhabdomyolysis, myoclonus, coarse tremors and cogwheeling. Common laboratory findings of NMS include increased CPK due to rhabdomyolysis, leukocytosis and myoglobinuria [8-12]. Some of these features were present in this patient and the authors believe this could be the cause for the reversible splenial lesion on MRI.

Another possibility that needs to be considered in this patient is hypernatremia. Maeda M et al reported a case of reversible splenial lesion in a patient with hypernatremia [13]. However, in this patient, there were also additional parenchymal lesions located symmetrically in the temporo-occipital lobes, which were not reversible and became more extensive in the follow-up MRI. This patient eventually developed osmotic myelinolysis and had a poor outcome. Although the clinical outcome in this patient was more favourable and there were no additional parenchymal lesions on MRI, hypernatremia as the cause for the reversible splenial lesion could not be entirely excluded.

The underlying pathophysiology of NMS is still poorly understood but it is thought to be caused by central and peripheral dopaminergic blockade that results in muscle rigidity, core temperature elevation and hypermetabolism. Virtually all classes of drugs that block the D_2_-receptors have been associated with NMS and it was found to be more frequent in patients receiving increasing and newly introduced doses of neuroleptic medication, medications given via intramuscular route, patients requiring physical restraints, patients suffering from mental disorders or retardation and those receiving a higher total dose of treatment [12]. Other risk factors include poor oral intake, dehydration, exhaustion, agitation and elevated temperature.

There are no specific neuroimaging features associated with NMS. CT Scan and MRI are usually done in patients with NMS to exclude other structural lesions or infections that may give rise to similar clinical presentation. Abnormalities in the corpus callosum have been reported in a patient with neuropsychiatric lupus with psychosis [14]. However, in this particular case, the patient also has history of seizures. The authors also concluded that the location of the lesion in the SCC associated with pure psychotic disorders raises the possibility that such a lesion may be sufficient to produce acute behavioral changes and psychotic features in certain patients.

To date, there has not been any reported case of reversible lesion of the SCC that is associated with NMS. Establishing a hypothesis for the precise etiology of the SCC lesion in this patient is difficult. The authors postulate that the lesion itself may be associated with the acute behavioral changes found in this patient. They also highlight the possibility of including NMS as one of the differential diagnosis of reversible splenial lesion of the corpus callosum.
